# Correlates of late-onset antipsychotic treatment resistance

**DOI:** 10.1192/j.eurpsy.2022.2018

**Published:** 2022-09-01

**Authors:** D. Fonseca De Freitas, D. Agbedjro, G. Kadra-Scalzo, E. Francis, I. Ridler, M. Pritchard, H. Shetty, A. Segev, C. Casetta, S. Smart, A. Morris, J. Downs, S. Christensen, N. Bak, B. Kinon, D. Stahl, R. Hayes, J. Maccabe

**Affiliations:** 1 King’s College London & University of Oxford College London, Psychological Medicine & Department Of Psychiatry And Nuffield Department Of Primary Care Health Sciences, London, United Kingdom; 2 Institute of Psychiatry, Psychology & Neuroscience, King’s College London, Psychological Medicine, London, United Kingdom; 3 Institute of Psychiatry, Psychology, and Neuroscience, King’s College London, Department Of Psychological Medicine, London, United Kingdom; 4 University College London, Institute Of Epidemiology And Health Care, London, United Kingdom; 5 Institute of Psychiatry, Psychology & Neuroscience, King’s College London, Biostatistics And Health Informatics, London, United Kingdom; 6 South London and Maudsley NHS Foundation Trust, Maudsley Biomedical Research Centre, London, United Kingdom; 7 Tel Aviv University, Sackler Faculty Of Medicine, Tel Aviv-Yafo , Israel; 8 Università degli Studi di Milano, Department Of Health Sciences, Milano, Italy; 9 Cardiff University, Mrc Centre For Neuropsychiatric Genetics And Genomics, Cardiff, United Kingdom; 10 King’s College London, Institute Of Psychiatry, Psychology & Neuroscience, London, United Kingdom; 11 H. Lundbeck A/S, H. Lundbeck A/s, Copenhagen, Denmark; 12 Lundbeck Pharmaceuticals LLC, Lundbeck Pharmaceuticals Llc, Deerfield, United States of America; 13 King’s College London, Academic Psychiatry, London, United Kingdom

**Keywords:** secondary TRS, refractory psychosis

## Abstract

**Introduction:**

There is emerging evidence of heterogeneity within treatment-resistance schizophrenia (TRS), with some people not responding to antipsychotic treatment from illness onset and a smaller group becoming treatment-resistant after an initial response period. It has been suggested that these groups have different aetiologies. Few studies have investigated socio-demographic and clinical differences between early and late onset of TRS.

**Objectives:**

This study aims to investigate socio-demographic and clinical correlates of late-onset of TRS.

**Methods:**

Using data from the electronic health records of the South London and Maudsley, we identified a cohort of people with TRS. Regression analyses were conducted to identify correlates of the length of treatment to TRS. Analysed predictors include gender, age, ethnicity, positive symptoms severity, problems with activities of daily living, psychiatric comorbidities, involuntary hospitalisation and treatment with long-acting injectable antipsychotics.

**Results:**

We observed a continuum of the length of treatment until TRS presentation. Having severe hallucinations and delusions at treatment start was associated shorter duration of treatment until the presentation of TRS.

**Conclusions:**

Our findings do not support a clear cut categorisation between early and late TRS, based on length of treatment until treatment resistance onset. More severe positive symptoms predict earlier onset of treatment resistance.

**Disclosure:**

DFdF, GKS, EF and IR have received research funding from Janssen and H. Lundbeck A/S. RDH and HS have received research funding from Roche, Pfizer, Janssen and Lundbeck. SES is employed on a grant held by Cardiff University from Takeda Pharmaceutical Comp

